# Case Report: Retropancreatic fascia hernia protruding into the thoracic cavity through a Bochdalek hernia

**DOI:** 10.3389/fped.2023.1149515

**Published:** 2023-06-26

**Authors:** Yoichi Nakagawa, Takuya Maeda, Hiroo Uchida, Shunya Takada, Akinari Hinoki, Chiyoe Shirota, Takahisa Tainaka, Wataru Sumida, Satoshi Makita, Hizuru Amano, Aitaro Takimoto, Yousuke Gohda

**Affiliations:** ^1^ Department of Pediatric Surgery, Nagoya University Graduate School of Medicine, Nagoya, Japan; ^2^ Department of Rare/Intractable Cancer Analysis Research, Nagoya University Graduate School of Medicine, Nagoya, Japan

**Keywords:** retropancreatic fascia, diaphragmatic hernia, Bochdalek hernia, retropancreatic fascia hernia, internal hernia

## Abstract

Retropancreatic fascia hernia is a novel internal hernia originating from the retropancreatic fascial defect, which subsequently expands toward the dorsal aspect of the pancreatic body and migrates into the retroperitoneal space. We encountered a rare case of concomitant retropancreatic fascia and Bochdalek hernias. Here, we describe the imaging characteristics of this hernia type and its surgical strategies.

## 1. Introduction

We previously reported a case of “retropancreatic fascia hernia,” which is a new type of internal retroperitoneal hernia originating from the retropancreatic fascial defect that passes through the dorsal aspect of the pancreatic body and enters the retroperitoneal space (1). Here, we report another case of retropancreatic fascia hernia with a concomitant Bochdalek hernia. Because of our awareness of this hernia type, preoperative diagnosis of the retropancreatic fascia hernia was possible, enabling the dispensing of appropriate surgical treatment. We report a rare case of retropancreatic fascia hernia and surgical treatment. This report was conducted in accordance with the SCARE guidelines (2).

## 2. Case description

**Figure 1:**
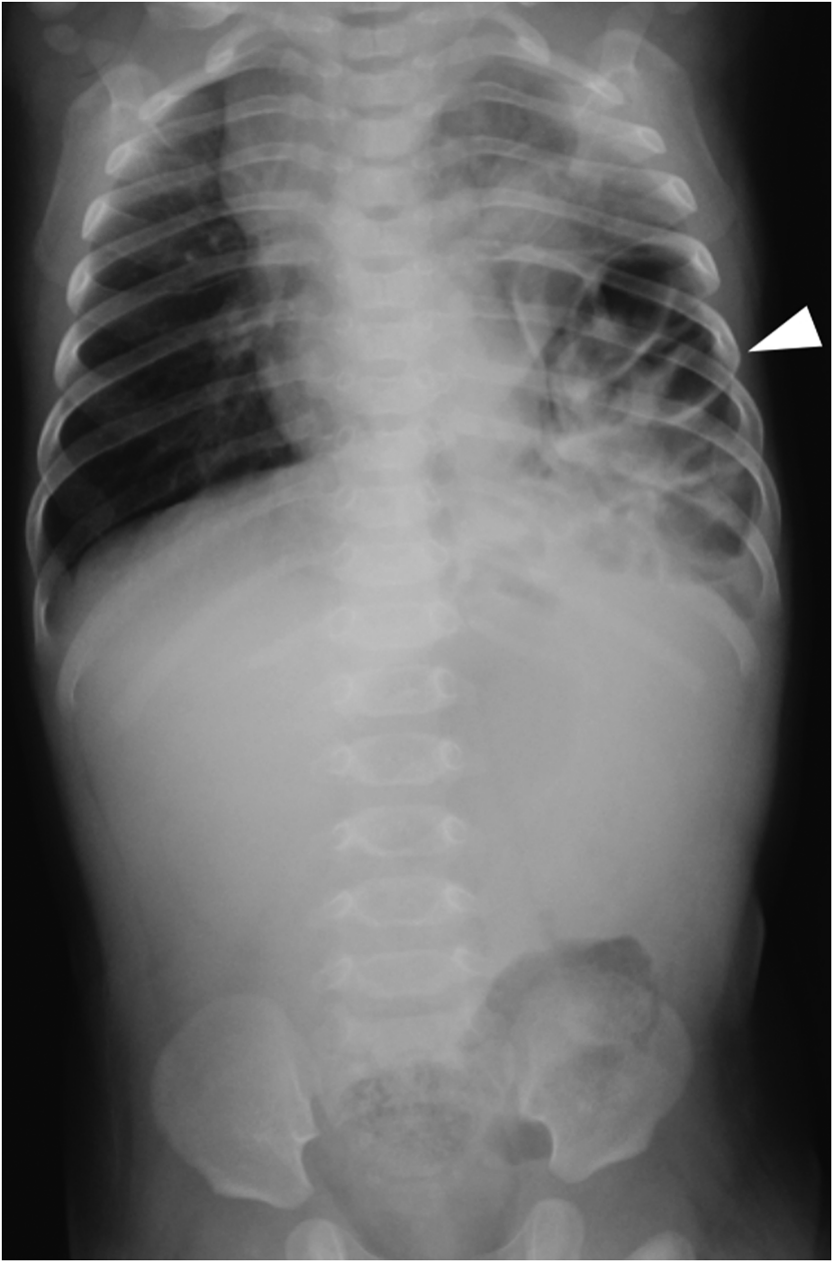
Preoperative chest and abdominal radiograph. A preoperative chest radiograph shows bowel gas in the left thoracic cavity.

**Figure 2:**
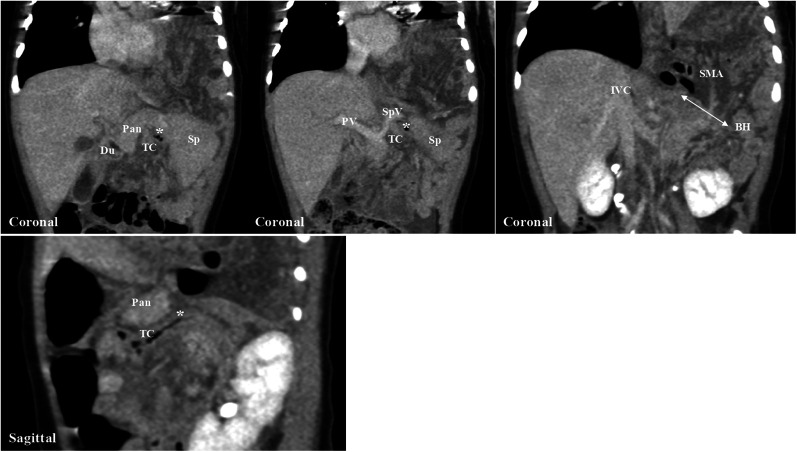
Preoperative computed tomography. Computed tomography shows the transverse colon and intestine protruding into the left thoracic cavity. The route of the internal hernia begins from the retropancreatic fascial defect, passes through the dorsal aspect of the pancreatic body, and enters the left thoracic cavity through the left Bochdalek foramen. Pan, pancreas; Du, duodenum; TC, transverse colon; Sp, spleen; PV, portal vein; SpV, splenic vein; IVC, inferior vena cava; SMA, superior mesenteric artery; BH, Bochdalek hernia.

**Figure 3:**
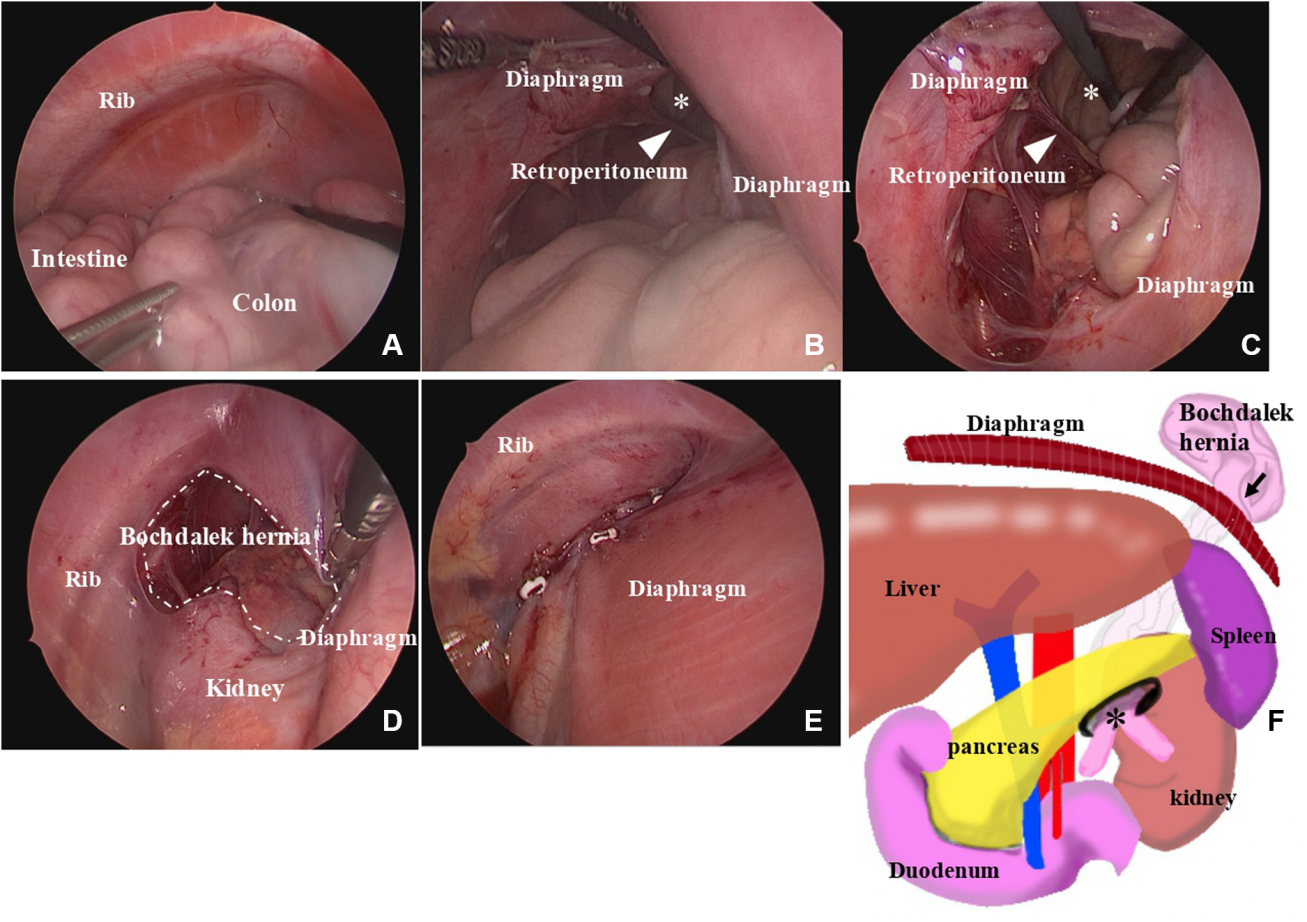
Intraoperative thoracoscopic findings. Thoracoscopic findings show that the intestine and colon are present in the thoracic cavity (**A**); the intestine and colon prolapse from the retropancreatic fascia hernia (*) into the Bochdalek hernia (**B**); the retropancreatic fascia hernia (*) is fully widened with scissors (**C**); the Bochdalek hernia is 30 mm in diameter (**D**); the defect hole is closed with 2-0 non-absorbable sutures (**E**); and the route of the retropancreatic fascia hernia into the Bochdalek hernia is shown as schema (**F**).

A 4-month-old Japanese male patient, who was delivered at a gestational age of 41 weeks without any perinatal abnormalities, presented with complaints of vomiting to our hospital. Prenatal ultrasonography did not detect any abnormalities in the patient. However, chest and abdominal radiography performed on admission showed bowel gas in the left thoracic cavity (Figure 1). A subsequent computed tomography (CT) revealed that the intestine and transverse colon had prolapsed through the orifice of the retropancreatic fascia hernia into the left thoracic cavity (Figure 2). A preoperative diagnosis of retropancreatic fascia hernia was made, and thoracoscopic surgery was performed. Intraoperative findings showed that the intestine and colon had prolapsed into the left thoracic cavity as a Bochdalek hernia (Figure 3A). However, we found a hole in the internal hernia (Figure 3B) through the foramen of Bochdalek. The intestine and colon prolapsed from the hole of the internal retropancreatic fascia hernia through the Bochdalek hernia; therefore, a retropancreatic fascia hernia with Bochdalek hernia was diagnosed. The internal hernial orifice was completely widened using scissors (Figure 3C) to reduce the herniated organs. After reduction, the Bochdalek hernial orifice was measured 30 mm in diameter (Figure 3D) and was closed by suturing the diaphragm and intercostal muscles with 2-0 non-absorbable sutures (Figure 3E). A schema of the hernia route is shown in Figure 3F. The retropancreatic fascia hernial orifice was not closed because the orifice was fully widened, and no risk of incarceration was observed. Finally, the patient was discharged on postoperative day 7 without any complications.

## 3. Discussion

Retropancreatic fascia hernia is a new type of internal hernia characterized by protrusion of the abdominal viscera through the retropancreatic fascia in the supramesocolic space. However, only two cases have been previously reported, one involving herniation into the posterior mediastinum and the other into the extrapleural space (1). Its treatment involves herniated organ reduction with either the closure or widening of the retropancreatic fascia defect hole. The previous two cases were treated by reducing the herniated organs and closing the opening of the pancreatic fascia hernia by performing laparotomy (1). In this case, the patient had a retropancreatic fascia hernia and a left Bochdalek hernia. The contents of the retropancreatic fascia hernia sac protruded into the thoracic cavity through the Bochdalek hernia. Generally, in a Bochdalek hernia, the intestinal tract and other organs protrude directly through the Bochdalek foramen; however, this is contrary to this case. These findings were apparent on preoperative CT and were intraoperatively confirmed.

In this case, a preoperative diagnosis of a retropancreatic fascia hernia with a diaphragmatic hernia was suspected based on imaging studies. Chest radiography showed a diaphragmatic hernia; therefore, a CT would have been unnecessary if the patient was a neonate. However, a CT examination was performed to confirm a late-onset diaphragmatic hernia because we have occasionally encountered atypical diaphragmatic hernias, as in this case. The thoracoscopic repair was selected over a laparoscopic approach because we believed that the herniated organs could be reduced by the insufflation pressure. Considering that a retropancreatic fascia hernia is an internal hernia, herniated organ reduction and hernial defect repair by simple closure or widening the sac opening are the treatment choices (3). Therefore, the laparoscopic approach for repairing the hernial orifice of the retropancreatic fascia would be preferred to the thoracoscopic approach. However, in this case, CT revealed a wide opening of the retropancreatic fascial defect as well as a large Bochdalek hernia; therefore, we opted for the thoracoscopic approach. In addition, widening the retropancreatic fascial defect was sufficient to treat the patient because the large Bochdalek hernia contributed to exaggerating the internal hernia in the case, and its repair prevented the recurrence. In this case, the retropancreatic fascial sac had a hole through which the intestine directly protruded into the thoracic cavity. Two previous reports have shown that retropancreatic fascia hernias protrude into the extrapleural space and posterior mediastinum (1) with their sacs. Although retropancreatic fascial hernia is a novel type of internal hernia, its treatment is yet to be determined. The hernia originates from the retropancreatic fascial defect; however, the hernial route could vary from case to case. Therefore, a detailed preoperative evaluation of the route taken by the retropancreatic fascia hernia is essential for its appropriate repair.

In conclusion, this is a rare case of retropancreatic fascia hernia with Bochdalek hernia. Thoracoscopic repair of Bochdalek hernia was successfully performed with a full widening of the internal hernial orifice. Although the clinical characteristics of retropancreatic fascia hernia vary, detailed preoperative imaging is critical for developing a surgical strategy.

## Data Availability

The original contributions presented in the study are included in the article, further inquiries can be directed to the corresponding author.

## References

[1] Nakagawa Y Uchida H Makita S Yokota K Hinoki A Shirota C A new type of retropancreatic fascia hernia in the supramesocolic space preoperatively misdiagnosed as a diaphragmatic hernia: report of two cases. Surg Case Rep. (2023) 9:5. 10.1186/s40792-023-01586-y 36627540 PMC9832205

[2] Agha RA Franchi T Sohrabi C Mathew G Kerwan A Thoma A The SCARE 2020 guideline: updating consensus surgical CAse REport (SCARE) guidelines. Int J Surg. (2020) 84:226–30. 10.1016/j.ijsu.2020.10.034 33181358

[3] Brigham RA Fallon WF Saunders JR Harmon JW D’avis JC. Paraduodenal hernia: diagnosis and surgical management. Surgery (1984) 96:498–502. 6474354

